# Behavioural impact of antibiotic stewardship in children in primary care: interviews with GPs and parents

**DOI:** 10.1093/jacamr/dlae207

**Published:** 2024-12-17

**Authors:** Erinn D’hulster, Marina Digregorio, Tine De Burghgraeve, Jeroen Luyten, Samuel Coenen, Sibyl Anthierens, Jan Y Verbakel

**Affiliations:** Department of Public Health and Primary Care, KU Leuven, Leuven, Belgium; Department of Family Medicine and Population Health, University of Antwerp, Antwerp, Belgium; Department of General Medicine, University of Liège, Liège, Belgium; Department of Public Health and Primary Care, KU Leuven, Leuven, Belgium; Department of Public Health and Primary Care, KU Leuven, Leuven, Belgium; Department of Family Medicine and Population Health, University of Antwerp, Antwerp, Belgium; Department of Family Medicine and Population Health, University of Antwerp, Antwerp, Belgium; Department of Public Health and Primary Care, KU Leuven, Leuven, Belgium

## Abstract

**Background:**

The ARON study, a randomized controlled trial, assesses a behavioural intervention incorporating clinically guided C-reactive protein (CRP) point-of-care testing and a parental information booklet to reduce inappropriate antibiotic prescriptions for acutely ill children in Belgian primary care.

**Objectives:**

To explore GP and parent views and experiences regarding the ARON trial intervention.

**Methods:**

We conducted a qualitative embedded process evaluation in Belgian general practice. Semi-structured interviews were held with purposively sampled GPs and a convenience sample of mothers of acutely ill children presenting to primary care. Data were analysed using inductive thematic analysis.

**Results:**

Thirty-four interviews were conducted with 17 GPs and 17 parents from the intervention arm, and four themes were identified. The first theme centres on the supportive role of CRP point-of-care testing in reducing diagnostic uncertainty and decreasing inappropriate prescriptions. The second theme explores the use of CRP in managing perceived parental expectations of antibiotics. The third theme discusses the use of intermediate CRP levels (above the trial’s 5 mg/L cut-off) as an indicator of serious infection, as opposed to its intended role in the trial as a rule-out factor. The final theme delves into the dual functionality of the booklet, enhancing self-management and offering reassurance through safety-netting advice. A logic model depicts the assumptions and (un)anticipated dynamics underlying the relationships between these themes and their subthemes.

**Conclusion:**

Both GPs and parents consider the intervention to be a helpful complementary tool during consultations for acutely ill children.

## Introduction

While the majority of childhood infections are typically self-limiting, and serious infections in children are rare,^1^ the estimated rate of antibiotic prescriptions for acutely ill children in ambulatory care remains high, at around 45% in high-income countries.^2^ Inappropriate antibiotic prescriptions for children in primary care pose a considerable problem, exacerbating the threat of antimicrobial resistance (AMR) and subjecting young patients to potential side effects.^3,4^

Despite increased awareness of this issue, inappropriate antibiotic prescribing remains highly prevalent due to various long-established social and emotional behaviours, such as GPs assuming parents/patients desire antibiotics, limited education of patients/parents, and diagnostic uncertainty experienced by GPs. Child-specific factors also contribute to a more risk-averse prescribing behaviour in the paediatric population, including clinicians’ perceptions of children as inherently vulnerable, and GPs’ negative assessments of parents’ ability to manage a child’s illness.^5^

To address these behaviours and optimize antibiotic prescribing practices in children, multifaceted behavioural interventions targeting both parents and clinicians during consultations are proposed as an effective strategy.^6,7^ In the context of adult patients in primary care, C-reactive protein (CRP) point-of-care testing (POCT) and patient information leaflets with safety-netting advice have demonstrated both clinical effectiveness and cost-efficiency.^8–10^ For children in primary care, the clinical effectiveness and cost-effectiveness of a multifaceted intervention comprising clinically guided CRP POCT and a parent information booklet on antibiotics including safety-netting advice is currently being investigated.^11^

The complexity of (multifaceted) interventions, assessed in controlled trial settings, frequently prompts concerns about their applicability beyond the research environment. It is essential to incorporate qualitative process evaluations, to achieve a more profound insight into the factors influencing the success and challenges of the intervention and to ensure an accurate interpretation of the quantitative trial outcomes.^12^ This comprehensive understanding, in turn, plays a crucial role in shaping effective implementation strategies.

To date, there has been a paucity of qualitative process evaluations of complex interventions designed to enhance antibiotic stewardship in primary care, especially within the context of children receiving primary healthcare services.^13^

In this study, we present a qualitative process evaluation conducted alongside a pragmatic clinical trial. Our objective was to examine the behavioural impact of an antibiotic stewardship intervention on antibiotic prescribing in acutely ill children and determine associated contextual factors that may affect the trial’s outcomes in a specific setting.

## Methods

This study is a nested qualitative process evaluation using individual semi-structured interviews with GPs and parents participating in the ‘Antibiotic prescribing Rate after Optimal Near-patient testing in acutely ill children in ambulatory care’ (ARON) trial. It adheres to the Standards for Reporting Qualitative Research (SRQR) and the Consolidated Criteria for Reporting Qualitative Research (COREQ) checklist.^14,15^

### Setting: the ARON trial

The ARON trial, a pragmatic cluster randomized controlled superiority trial, examines the clinical effectiveness and cost-effectiveness of a diagnostic algorithm in safely reducing antibiotic prescribing for acutely ill children aged 6 months to 12 years in ambulatory care. The trial enrolled 6750 children from primary care or community paediatric practices in Belgium.

**Figure 1.**
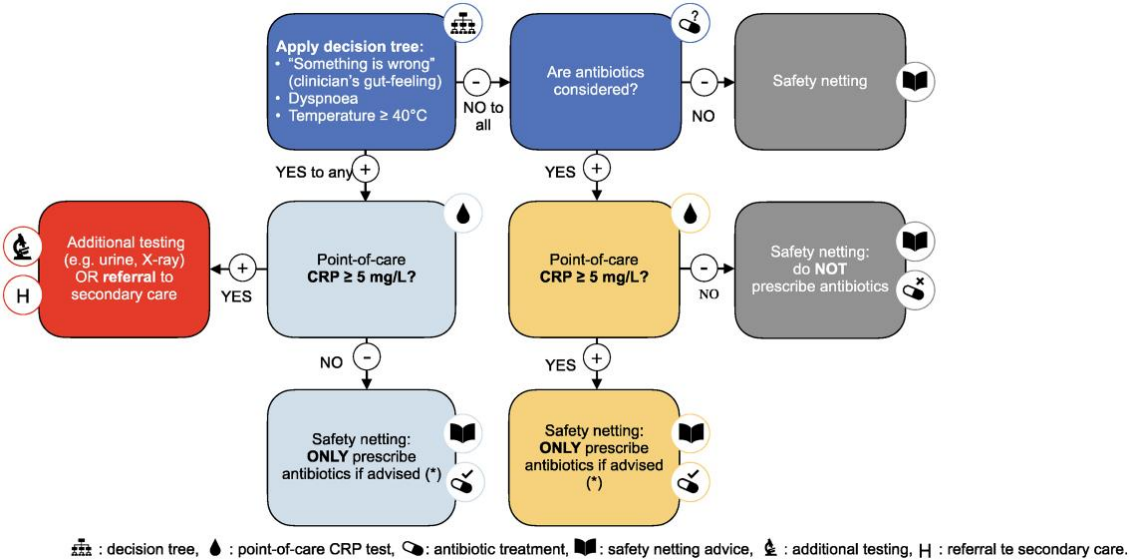
Detailed flowchart followed in intervention arm in ARON trial. (*), according to guidance on antibiotic prescribing (BAPCOC guide). Reproduced with permission from Verbakel *et al*.^11^

The multifaceted algorithm studied in the ARON trial combines clinically guided CRP POCT and an evidence-based parent information booklet with safety-netting advice (detailed in Figure 1).^13,16,17^ If the CRP level is 5 mg/L or higher, referral or additional testing could be considered to rule out a potential serious infection.

The complete ARON trial protocol, registered under NCT04470518 on clinicaltrials.gov, can be found elsewhere.^11^

### Recruitment and sampling

Both GPs and parents of children participating in the intervention group were interviewed. The GPs were purposively sampled to achieve maximum variation across various parameters, including age, gender, practice region (rural or urban), practice type, number of CRP tests performed, and recruitment rate.

For parents, we compiled a convenience sample exclusively including those whose children had been enrolled in the ARON trial by a clinician who was also interviewed.

### Interviews

Interviews were conducted by two female researchers trained in qualitative interviewing (E.D. and M.D.). E.D. has a background in health economics and M.D. has a biomedical background. Both interviewers were well acquainted with the trial design and content, drawing from their roles as study coordinators in the ARON trial.

GPs were interviewed during their participation in the trial, while parents of patients were interviewed within 30 days following the index consultation to mitigate recall bias. All participants provided digital consent.

A topic guide was used to ensure key areas were covered, with the flexibility to allow participants to raise issues that were personally important. The guide for clinicians was informed by the overall aim of the qualitative study (see Table S1, available as Supplementary data at *JAC-AMR* Online) and underwent review by all study team members and was pilot-tested with a GP not involved in the study. It explored various topics, including general impressions of study participation and perspectives on the use of the CRP test and booklet, with a primary focus on understanding the behavioural impact of the intervention. For parents, the topic guide was piloted with a mother of two young children who did not participate in the study. Topics covered the reason for consulting, the general impression of the consultation, and views on and experiences with the booklet and CRP testing, with a primary emphasis on the latter.

Data collection and analysis occurred concurrently. Data collection stopped when no new themes were being identified or significantly elaborated.^18^

We planned to interview 14–18 participants in each group.

### Analysis

All interviews were audio-recorded, then transcribed verbatim, checked and anonymized by E.D.

An inductive thematic analysis approach was employed that ensured the organic emergence of themes from the original data.^19^ Clinicians’ and parents’ data were analysed separately by two authors using a combination of deductive and inductive thematic analysis. The initial step of familiarization was conducted by E.D., who thoroughly reviewed all data, while S.A. (social scientist) read a subset of interviews. At each stage of the analysis process, the potential themes were actively constructed using a comprehensive codebook through collaborative discussions among J.V. (GP), S.A. and E.D. to ensure rigour. The resulting thematic framework was then used to code the remaining interviews. Data triangulation was used by comparing clinicians’ and parents’ datasets to further understand key similarities and differences in relation to the study aim.

To enhance the quality of the analysis, researcher triangulation was carried out through discussions of the data among the wider multidisciplinary team. QSR NVIVO software version 12 (QSR International Pty Ltd, Melbourne, Australia) was used to support data analysis.

### Ethics

The study protocol and associated documentation received approval from the Ethics Committee Research of University Hospitals Leuven under reference S62005.

## Results

Interviews took place between February 2022 and February 2023, with an average duration of 44 min for clinicians and 37 min for parents.

A total of 17 GPs and 17 parents (only mothers could be recruited) were interviewed for the study. The participants’ characteristics are detailed in Table 1.

**Table 1.** Characteristics of GPs and parents involved in the process evaluation of the ARON trial

GP characteristics (*N* = 17)		*n*	Parent characteristics (*N* = 17)		*n*
Age, years	20–30	4	Age of child	6 months–2 years	6
	>30–40	9		>2–5 years	4
	>40–50	2		>5–8 years	4
	>50	2		>9–12 years	3
Gender	Female	12	Gender of child	Female	7
	Male	5		Male	10
Language	Dutch	13	Language	Dutch	13
	French	4		French	4
Years of medical experience	≤5	4	Gender of parent	Female	17
	>5–10	6		Male	0
	>10–20	4	Number of children in total	1	9
	>20–30	1		2	6
	>30	2		3	2
Practice region	Rural	5	CRP test done?	Yes	5
	Urban	12		No	12
Practice type	Solo	2	Antibiotics received?	Yes	3
	Duo	4		No	14
	≥3 GPs (group)	11			
Use of CRP device, %	0–25	5			
	26–50	8			
	51–75	4			
Prescription of antibiotics, %	0–15	6			
	16–30	7			
	31–45	4			

**Figure 2.**
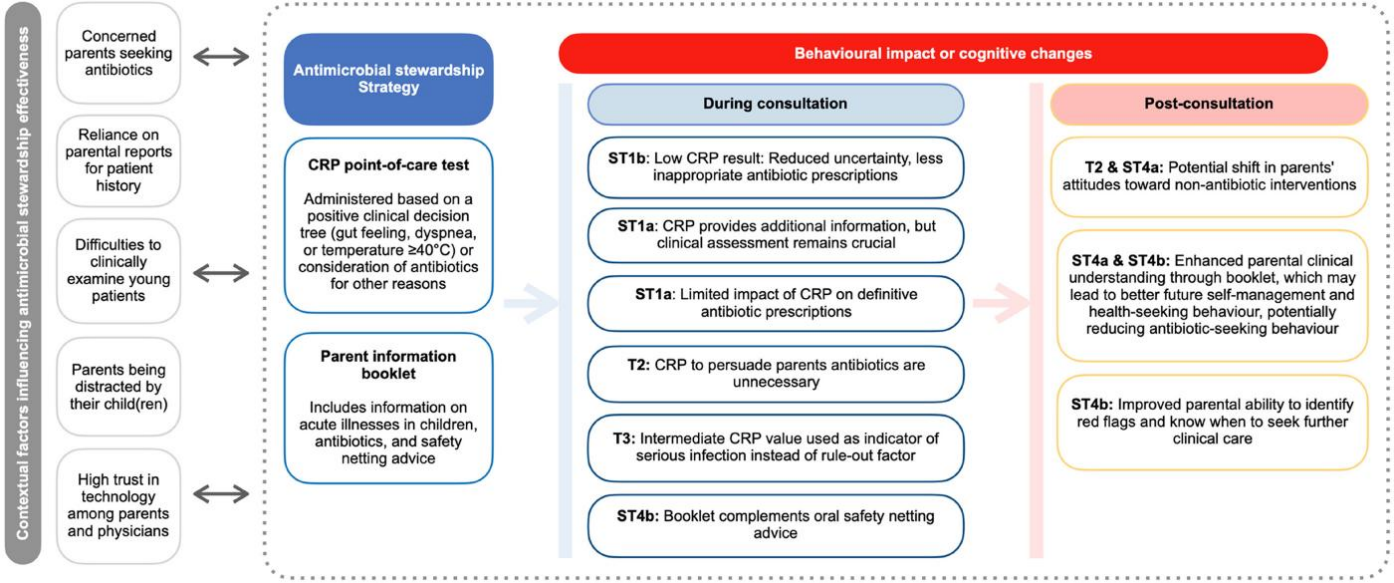
Comprehensive logic model referring to main themes (T) and subthemes (ST). Anticipated causal assumptions and unexpected dynamics emerging from the interviews.

We identified four main themes, primarily focusing on the behavioural impact of the intervention, aligning with the main objective of this study. The relationships between these themes are depicted in a logic model (Figure 2).

After each quote from a clinician, the percentage of patients for whom the clinician conducted a CRP test and the percentage of included patients for whom the clinician prescribed antibiotics are provided. Similarly, following each quote from a parent, the age of the child in the ARON study, the antibiotic prescription decision and whether a CRP test was administered are indicated.

### Theme 1: Supportive role of CRP result in reducing diagnostic uncertainty and decreasing inappropriate prescriptions

#### Subtheme 1a: CRP as an additional argument alongside clinical judgement in antibiotic decision-making

The majority of GPs emphasized the utility of CRP POCT, highlighting its supportive role in reducing diagnostic uncertainty. Clinicians stated that CRP results provide additional information, serving as an objective argument influencing their decision-making process.

There was a consensus among the interviewed GPs that integrating CRP testing into their diagnostic approach was particularly beneficial in cases characterized by high diagnostic uncertainty. Diagnostic uncertainty, stemming from factors such as ambiguous guidelines or incomplete patient history, can prompt a more defensive approach in antibiotic prescribing. In this context, CRP testing serves as a valuable tool, offering a means to reduce uncertainty and enable more appropriate prescription practices.

‘*Following a failsafe principle, when you're unsure about diagnoses, you treat the worst-case scenario you’re uncertain about. This might mean antibiotics get prescribed more quickly. And I believe there, the CRP test can significantly help in prescribing antibiotics more selectively.*’ [*Clinician 5, male, CRP in 34%, antibiotics in 16%*]

Contrary to this, CRP does not significantly contribute to decision-making when clinicians are firmly convinced that antibiotics are necessary based on clinical judgement. In such cases, a CRP result is unlikely to have any impact on the prescribing decision.

*If the ear infection is bilateral and the child is, let’s say, 1 year old, then I would start antibiotics according to the guidelines. Now, I do the test because it’s required for the study, but honestly, I don't see CRP as an added value in those cases because it won't change my approach.*’ [Clinician 6, female, CRP in 34%, antibiotics in 24%]

According to both GPs and parents, the decision to prescribe antibiotics remains heavily reliant on clinical judgement. While acknowledging the role of CRP in the decision-making process, clinical assessment retains substantial importance.

‘*Yesterday, I had someone with a CRP of 7. Still, I put them on antibiotics because the clinical context justified it. So, it’s just one element among the others.*’ [Clinician 4, male, CRP in 13%, antibiotics in 11%]

#### Subtheme 1b: Reassurance from low CRP result in cases of uncertainty

All GPs noted that, when uncertain about the need for antibiotics, obtaining low CRP results, especially those falling below the 5 mg/L trial cut-off, provides reassurance for both healthcare providers and parents. These results instil confidence in clinical decision-making, facilitating a ‘wait and see’ approach.

#### Subtheme 1c: Diagnostic challenges in small children and CRP’s support

Most GPs emphasized the practical value of CRP in young patients because of the diagnostic challenges they pose. These complexities include the necessity of depending on parental reports for patient history and the difficulties they experience when clinically examining young patients.

‘*I really saw the benefit of using CRP in children. Since they can’t express well where the problems are, it’s harder to figure out what’s going on. But mainly because they’re more challenging to assess clinically. They’re not as easy to examine. They can*’*t describe their symptoms as well: ‘Where does it hurt?*’ [Clinician 2, male, CRP in 23%, antibiotics in 23%]

From the interviews, it also emerged that GPs anticipate that this finding regarding the additional value of CRP in children may be extended in the future to other patients who experience communication barriers, such as non-native speakers or individuals with limited medical knowledge.

### Theme 2: Use of CRP to manage parents’ perceived expectations of antibiotics

Several GPs noted that the supplementary information provided by CRP is effective in persuading parents in situations where the GP perceives that parents expect an antibiotic prescription, reinforcing their clinical judgement with an objective measure.

‘*Take those parents asking for antibiotics, maybe because of a sore throat or whatever. Showing them the result got really through to them, like, ‘Look, you don’t really need it. It’s not necessary.*’ [Clinician 8, female, CRP in 70%, antibiotics in 25%]

However, it is essential to note that not all parents were equally persuaded by a low CRP result. One parent expressed a lack of conviction in the test outcomes, stating:

‘*I was so convinced that there was something serious going on with my daughter that I didn’t really attach much importance to the test result. I just wanted antibiotics. I was like: just prescribe it, and I’ll go get it.*’ [Parent 1, female, CRP test, no antibiotics, 11-year-old daughter]

### Theme 3: Intermediate CRP result as indicator of serious infection instead of rule-out factor

In the ARON trial, in situations of diagnostic uncertainty and when the CRP result fails to rule out the possibility of a serious infection (i.e. CRP is not under 5 mg/L), clinicians are directed to consult the Belgian Commission for the Coordination of Antibiotic Policy (BAPCOC) guidelines for antibiotic prescription decisions. These guidelines do not specifically address or incorporate CRP values into their recommendations.

However, interviews revealed that in these situations some clinicians struggle to disregard CRP and solely adhere to the guidelines. Instead, they attempt to interpret CRP in relation to the presence or absence of a serious infection. Contrary to the study design—where CRP is intended to rule out severe infections due to its higher specificity but lower sensitivity—they use CRP to rule them in. In line with the anticipated adherence to guidelines in such instances, the ARON study does not provide guidance for clinicians in interpreting intermediate values, simply due to lack of an evidence base for these intermediate CRP levels.

‘*Sometimes you get these non-values like recently, I had a CRP of 30 in a child, and that’s not clear, you know. That’s very frustrating because it’s not very high, and not low either. I was hoping it would be less than 5; that would have put my mind at ease. I ended up prescribing antibiotics because there was clearly an elevation, though it was a delayed prescription.*’ [Clinician 10, female, CRP in 44%, antibiotics in 18%]

### Theme 4: Dual role of parental booklet—enhancing parents’ self-management and addressing concerns through safety-netting advice

#### Subtheme 4a: Strengthening parental self-management and health-seeking behaviour

While not all parents engage with the booklet, those who did report an improvement in their ability to manage and care for their child’s health. Parents with multiple children expressed less perceived benefit from the booklet, citing prior experience.

‘*The clinician showed the duration of different illnesses in the booklet, and when I learned that coughing could last quite a while, I thought, ‘Oh, okay, so it’s normal that my little one has been coughing for more than a week.’ It did reassure me a bit, actually.*’ [Parent 6, female, no CRP test, no antibiotics, 2-year-old daughter]

#### Subtheme 4b: Booklet as reference material for safety-netting, enhancing clinician–parent communication and providing mutual reassurance

Clinicians find the booklet useful as a tool to complement their safety-netting advice given during the consultation, ensuring that parents have comprehensive information.

‘*I think the parental booklet is quite handy to have. Sometimes, I see parents who come in for the first time, jotting down all the warning signs. And then it’s indeed helpful to have a booklet to give them, with everything listed, so they can just take it with them and read it at home.*’ [Clinician 13, female, CRP in 29%, antibiotics in 13%]

Parents value having the booklet as reference material as well, to revisit or reinforce their understanding of the discussed topics. They find it particularly helpful in the context of visiting with a sick child, as distractions such as crying child or one seeking attention by climbing onto their lap, can make it hard for parents to absorb information during consultations.

‘*I don’t remember very well what the clinician said about when I should come back. It’s possible he did mention it, but my son was constantly seeking attention, so I couldn’t fully concentrate on what the clinician was saying.*’ [Parent 8, female, no CRP test, no antibiotics, 7-year-old son]

By enhancing safety-netting communication, the booklet provides reassurance to both clinicians and parents. This may support a ‘wait and see’ strategy and lead to a reduction in antibiotic prescribing.

‘*I once lost a patient because the mother waited too long to come, and I’ve had this fear since then that parents might not assess the situation very well. And I think I might also prescribe antibiotics a bit sooner since then… And with the booklet, they can read that information again, which brings me a sense of relief.*’ [Clinician 10, female, CRP in 44%, antibiotics in 18%]

## Discussion

### Summary of findings

CRP is recognized as a valuable diagnostic tool, particularly in situations of diagnostic uncertainty. When the CRP result is low, GPs express confidence in refraining from prescribing antibiotics. Nonetheless, both GPs and parents emphasize the sustained significance of clinical judgement in the management of acutely ill children. CRP is not perceived as a mere substitute but rather as a valuable complement to clinical judgement, enhancing the overall decision-making process.

Conversely, the perceived utility of CRP testing diminishes when clinicians are confident in their decision to prescribe antibiotics.

Moreover, the CRP test is occasionally strategically employed to persuade parents that antibiotic treatment is unnecessary.

Some clinicians mentioned that in the case of diagnostic uncertainty and the CRP level falling in an intermediate range, they found it challenging to strictly adhere to established guidelines as directed by the protocol. Instead, they tended to shape their clinical decisions based on the CRP value, introducing interpretational challenges.

Finally, the parent booklet is also highly valued, serving dual purposes as an educational reference on acute infections in children for parents and as a resource for safety-netting advice. It guides parents on when to seek medical advice again in both current and future illness episodes. Clinicians also find reassurance in the booklet, feeling confident in delivering comprehensive information to parents, with the assurance that the safety-netting advice is well received as parents take the booklet home and have the opportunity to revisit it.

Clinicians deemed the CRP tests and the parental booklets to be complementary, as CRP proved beneficial during the consultation, while the booklet served its utility mainly post-consultation.

### Strengths and limitations

We encountered challenges in interviewing male parents, potentially introducing a gender bias. However, this corresponds with typical consultation patterns, where mothers frequently accompany their sick children to the clinician.

Moreover, the interviews were conducted by E.D. and M.D., both engaged as study coordinators in the ARON trial. Particular emphasis was placed on conveying that participants’ feedback, whether positive or negative, was crucial for a comprehensive evaluation of the trial. Additionally, questions were phrased in a non-leading manner to encourage participants to express their genuine experiences and opinions.

Finally, the expression of pre-existing dedication to combating AMR by some GPs during the interviews could influence the trial’s outcomes. The participation of clinicians with a strong commitment to appropriate antibiotic use may limit the transferability of our findings, as we may have less insight into clinicians on the opposite end of the spectrum in terms of dedication to combating AMR.

### Comparison with existing literature

Existing literature on this topic primarily focuses on single-component interventions like CRP testing or the use of an information booklet alone, or explores individual perspectives.^13,17,20–23^ They also frequently centred on aspects such as usability or accessibility of interventions, all with very limited consideration for the context of children.

Nevertheless, the process evaluation of the GRACE INTRO study examined both the booklet and CRP testing within primary care in patients and clinicians.^24,25^ Our results align with their findings. However, in that particular study, the booklet was part of a broader communication training programme for clinicians and the focus was mostly on adults.

Our study results generally align with prior qualitative research on CRP POCT in primary care,^20,21^ reporting the positive reception of CRP testing by parents, children and GPs, highlighting its utility in reducing diagnostic uncertainty and assisting GPs in managing patients’ expectations regarding antibiotic treatment. Additionally, our findings resonate with a study by Lemiengre *et al*.,^26^ indicating that some clinicians face challenges in disregarding CRP and strictly adhering to guidelines when confronted with intermediate CRP values. However, most of these studies on CRP testing primarily centre on adults, given its established clinical effectiveness in this demographic.

A study by Schot *et al*.,^22^ focusing on CRP in children in primary care, presented results inconsistent with our findings. The authors reported a reluctance among GPs to integrate CRP POCT into the diagnostic evaluation of children, citing reasons such as the invasiveness of the test and the vulnerability of children. These concerns were not raised by GPs interviewed in this process evaluation. We should note that the study by Schot *et al*. was conducted in a Dutch context characterized by conservative antibiotic practices, which could explain the disparity in results.

Additionally, results from existing qualitative studies on parent information booklets conducted in healthcare systems different from the Belgian context align with our findings.^13,17^ This agreement is not entirely coincidental, as the booklet used in the ARON trial is inspired by the ones examined in those qualitative studies.

Finally, our study findings complement those of Dillen *et al*.,^27^ who identified physician-related factors, such as rural practice settings and GP prescribing habits, as contributors to inappropriate prescribing. In addition, we found patient-level factors, such as diagnostic uncertainty and the difficulty of interpreting intermediate CRP values, some of which are specific to children, like perceived parental expectations and the diagnostic challenges associated with young children. These child-specific factors resonate with Cabral *et al*.’s research,^5^ which highlights how the social construction of child vulnerability may drive defensive prescribing in paediatric care. Furthermore, our study shows how these factors and their interplay can potentially be addressed through a multifaceted stewardship approach.

### Implications

Our findings suggest that clinicians, outside the study context, are likely to primarily employ CRP testing in situations characterized by diagnostic uncertainty, considering it as a valuable source of additional information. However, it is less likely that CRP will be employed when there is a strong initial inclination to prescribe antibiotics.

Furthermore, when using CRP as a persuasive tool to manage parental expectations for antibiotics, clinicians should avoid presenting CRP as an absolute argument that dismisses deeply held parental beliefs. Instead, the test result should be incorporated into a nuanced approach that emphasizes open communication with the parents.

Additionally, clinicians’ challenges in interpreting intermediate, so-called ‘grey zone CRP values’, highlight the need for clearer guidance. Ideally, strategies will be implemented to harmonize the interpretation of CRP within established guidelines to ensure consistent decision-making.

The favourable reception of the information booklet suggests its potential for broader implementation, with the possibility of online distribution to enhance accessibility.

Although our study was conducted in Belgian primary care, the findings may offer transferable insights that could be applied to similar settings with appropriate adaptations. Transferability recognizes that findings can be relevant in different contexts, but only when the similarities and differences between the original and new settings are carefully considered.

In low- and middle-income countries (LMICs), where AMR poses a significant threat,^28^ adapting our intervention would require attention to local factors such as healthcare infrastructure, economic constraints and cultural perceptions of antibiotic use. For instance, while the parental information booklet could be beneficial in LMICs where accessible educational tools are crucial, its successful implementation would depend on factors like health literacy levels, access to healthcare, and the specific dynamics of antibiotic use in these regions.

## Supplementary Material

dlae207_Supplementary_Data

## Supplementary data

Table S1 is available as Supplementary data at *JAC-AMR* Online.
