# Supplementary material for: Behavioural impact of antibiotic stewardship in children in primary care: interviews with GPs and parents
Source: JAC Antimicrob Resist. 2024 Dec 17;6(6):dlae207. doi: 10.1093/jacamr/dlae207 (PMC11649757; doi:10.1093/jacamr/dlae207)
Supplement: dlae207_Supplementary_Data [file dlae207_supplementary_data.docx]

**SUPPLEMENTARY DATA**

**Table S1 – Topic guide** (Available in French and Dutch on request)

| **ARON Trial – Topic guide (Clinicians)**    **INTRODUCTION**    1. Thank the participant for participating in this interview. 2. Introduce yourself. 3. Explain that this interview is part of the ARON trial and that the goals of the interview are described in the participant information sheet. 4. Explain that it is not mandatory to answer a question if one is not comfortable doing so. 5. Ask if it is ok to audio and video record the conversation. The recording will be transcribed, but data will be pseudonymized. Communicate that names and place names will never be noted so that someone reading the transcript will never be able to figure out who is sitting in front of you. 6. Inform the participants that if he/she wants the recording to stop, he/she may indicate this at any time. 7. Ask if the participant has any questions. 8. Forward the link to the participant by email to complete the online informed consent form via Qualtrics. The informed consent form will be completed together with the investigator.     **TOPICS TO BE EXPLORED**     - Participants’ views and experiences of using the diagnostic algorithm including POC CRP testing (e.g., perceived barriers and facilitators to using the diagnostic algorithm). - Factors that influence the behavior to prescribe antibiotic treatment whilst taking part in the intervention arm (e.g., awareness of antimicrobial resistance issues). - Physician’s views and experiences of taking part in the trial.     **EXAMPLE QUESTIONS**     - **Introduction**      - How did you experience participating in the ARON Trial? - Were you well supported by the study team? How did you experience setting up the trial in your practice? - Why were you interested in participating in this clinical trial? - Were there any facets of the study that you especially liked? Could you clarify why/why not? - How did you experience taking care of patients using the diagnostic algorithm of the ARON Trial?      - **Antibiotic prescribing in children**      - What are your thoughts on antimicrobial resistance? - Do you sometimes/frequently worry about the use of antibiotics? - What factors influence your prescribing behavior in sick children? - In what way is your prescribing behavior different with a child? In what way do parents have an influence (or not) on your prescribing behavior? When do you use the guidelines or how do you use the guidelines? If not, why not? - Has participating in the intervention changed your management of acutely ill children? - Why? Why not? - **Diagnostic tests (POC CRP test)**      - How did you experience the use of CRP tests in primary care? - Did you find it helpful? Why/why not? Were there specific patients or certain situations where you found it particularly useful? Have you experienced any cases where the test results did not correspond to your clinical findings? If so, what did you rely on? - What do you think are the advantages of this test? - Did CRP testing have an impact on your antibiotic prescribing rate? Why/why not? Did the test make you feel more confident about your diagnosis or treatment plan? - In your opinion, what are the (potential) disadvantages of POC CRP tests in primary care? - Was waiting for a test result during the consultation tedious? Was there a steep learning curve to work with the device and/or interpret the results correctly? - What do you think your patients and their parents thought of this diagnostic test? - Were some parents reluctant to have their child's finger pricked? Did the test result help you to communicate your treatment plan? if so, how? How did you communicate the test result to the parents and/or patients? How did parents respond? Did you ever decide not to use the test even though the diagnostic algorithm recommended it? In what way did the use of the CRP test affect your relationship with your patients? - How do you feel about future use of the CRP device outside the scope of this trial? - What needs to be provided so that you will use the tests daily? Are there any facilitators (e.g., financial compensation for the physician, reimbursement for the test, inclusion in guidelines)? Are there any barriers that need to be addressed?      - **Safety netting advice (booklet)**      - How did you experience the use of safety netting booklets? - Did you find it helpful? Why/why not? Were there specific patients or certain situations where you found it particularly useful? - What do you think (the parents of) your patients thought of this booklet? - Did you go over the booklet together with the parent(s) and the patient? What factors influence how you communicate this advice to parents? Do you feel that the parents understand this advice? Why or why not? - What do you think are the advantages of this booklet? - Did the booklet have an impact on your antibiotic prescribing rate? Why/why not? Did the booklet make you feel more confident about your treatment plan? - In your opinion, what are the (potential) disadvantages of safety netting booklets in primary care?      - **Concluding questions**      - If there is anything else you would like to add, please go ahead. - Do you have a preference between having a finger prick test in a consultation or handing out a booklet? If so, why is this? - What do you think is an acceptable price for a CRP test? - How many minutes does it take to do a CRP test?     **ENDING OF THE INTERVIEW**    Thank you very much for your time. |
| --- |

| **ARON Trial – Topic guide (Parents)**    **INTRODUCTION**    1. Thank the participant for taking part in this interview. 2. Introduce yourself. 3. Explain that this interview is part of the ARON trial and that the goals of the interview are described in the participant information sheet. 4. Explain that it is not mandatory to answer a question if one is not comfortable doing so. 5. Ask if it is ok to audio and video record the conversation. The recording will be transcribed, but data will be pseudonymized. Communicate that names and place names will never be noted so that someone reading the transcript will never be able to figure out who is sitting in front of you. 6. Inform the participants that if he/she wants the recording to stop, he/she may indicate this at any time. 7. Ask if the participant has any questions. 8. Forward the link to the participant by email to complete the online informed consent form via Qualtrics. The informed consent form will be completed together with the investigator.     **TOPICS TO BE EXPLORED**     - Parents’ expectations and whether they were met during the consultation. - Parents’ views and experiences of undergoing the diagnostic algorithm including POC CRP testing (e.g., perceived barriers and facilitators). - Parents’ views and experiences of taking part in the trial (including the follow-up period).     **EXAMPLE QUESTIONS**     - **General consultation (Introduction)**      - How did you experience the consultation with the doctor on the day that you and your child agreed to participate in the ARON Trial? - What was the reason you sought help from a GP? Were your concerns addressed by the GP? Did this consultation last longer than usual? Was anything different during the consultation from previous consultations? What did you like about it and what did you not like about it? - Why were you interested in participating in this clinical trial?      - **Treatment**      - Did the doctor prescribe antibiotics for your child? If so, did the doctor advise you to administer them immediately or to wait until the symptoms got worse? - Did the treatment meet your expectations? Why or why not? How did you perceive the proposed treatment by the doctor? How did you feel about it? - Was your child prescribed medication that is not an antibiotic? If so, how did it make you feel? - Do you know what antimicrobial resistance is? (If not, explain to participant) - Do you consider this issue when your child is prescribed antibiotics?      - **Diagnostics**   CRP test     - Do you remember a test being carried out on your child which required the doctor to prick his/her finger? If so, what are your thoughts on this test? - Were you reluctant due to the finger prick? What did the GP say about this test? - Do you remember the results of the test? - Were the results carefully discussed? Did you understand the purpose of the test? Do you think the test result has led to the treatment suggested by the doctor? If so, did you understand the link between the results and the treatment plan? - Did you perceive any advantages and/or disadvantages of this test? - Does the test make you less or more likely to consult a GP in the future for a similar problem? Please explain.   Additional examinations   - Did your child have to undergo one or more (additional) tests (e.g., saturation measurement, urine test…)? If so, how did you experience the additional testing? - Was the reason for this additional examination well communicated by your GP? Did you understand the necessity? - **Safety netting advice**    Booklet - Do you remember receiving a booklet describing possible alarm symptoms? - Did you go over this booklet with the GP and/or read it yourself at home? If so, how did you perceive this booklet? - Did this booklet have a reassuring effect, or did it make you anxious about your child's health? Did you perceive any advantages or disadvantages of this booklet? What can be improved about the booklet? - Does the booklet make you less or more likely to consult a GP in the future for a similar problem? - **Follow-up: Smartphone app (QoL)** - Did you fill in the smartphone app after the consultation, in which the health of your child was assessed? If so, how did you experience this? - Did you find the app user-friendly to fill in? Did the app accurately capture your child's health?      - **Concluding questions**      - If there is anything else you would like to add, please go ahead. - (Only for intervention group) Do you have a preference between having a finger prick test in a consultation or receiving a booklet? If so, why is this?     **ENDING OF THE INTERVIEW**    Thank you very much for your time. |
| --- |
